# Postmortem radiological case series of acetabular fractures after fatal aviation accidents

**DOI:** 10.1007/s12024-018-9946-1

**Published:** 2018-02-05

**Authors:** Henri M. de Bakker, Melanie Tijsterman, Bela Kubat, Vidija Soerdjbalie-Maikoe, Rick R. van Rijn, Bernadette S. de Bakker

**Affiliations:** 1 Department of Radiology, Groene Hart Hospital, Gouda, The Netherlands; 2 Department of Medical Biology, Section Clinical Anatomy and Embryology, Academic Medical Center, University of Amsterdam, Meibergdreef 15, 1105 AZ Amsterdam, The Netherlands; 3 Division of Special Services, Section Forensic Pathology, Netherlands Forensic Institute, The Hague, The Netherlands; 4 Department of Pathology, Maastricht University Medical Center, University of Maastricht, Maastricht, The Netherlands; 5 Department of Radiology, Academic Medical Center, University of Amsterdam, Amsterdam, The Netherlands

**Keywords:** Aviation accidents, Postmortem, Acetabulum, Acetabular fracture, Forensic radiology

## Abstract

The purpose of this study was to describe radiological fracture patterns of the acetabulum sustained after fatal small aircraft aviation accidents, aiming at facilitating a better understanding of trauma mechanisms in a forensic setting. Postmortem conventional radiographs or CT scans of 29 victims of 20 small aircraft aviation accidents were analyzed for skeletal acetabular trauma. Among the 29 fatalities (27 males and 2 females, median age 55 years (range: 21–76 years)), 20 victims had pelvic fractures (69%), of which 19 victims (66%) had one or more acetabular fractures. Bilateral acetabular fractures occurred in 11 victims. When considering left and right acetabula in each victim as separate entities, 38 of the 58 acetabula included in this case series exhibited one or more fractures. Both the anterior and posterior acetabular columns were fractured in 28 acetabula. Acetabular fractures were frequently encountered in this series of 29 victims of small fatal aircraft accidents. Fractures of the acetabulum occur from ventrally directed impact (i.e. to the knee) or laterally directed impact (i.e. to the greater trochanter of the femur). Radiological descriptions of the fracture patterns can therefore aid in the forensic analysis of the mechanism of trauma in aviation accidents. Postmortem multi-slice CT scan images are preferrable in the assessment of acetabular fractures.

## Introduction

In a forensic setting, the use of radiological imaging as an addition to conventional autopsy has increased in recent decades. Postmortem computed tomography (PMCT) has proven to be a helpful tool in the quest for the legal truth, as it contributes to the visualization of skeletal traumatic lesions and is helpful in the determination of the cause and reconstruction of the manner of death [1, 2]. For this reason, aviation accidents are forensically interesting. Various studies have described injuries obtained during aviation accidents [3–9]. As was suggested by Kubat et al., the type and severity of injuries depend on many factors [7, 8], such as the speed of the aircraft, the type of the aircraft, or the position of the persons in the plane. Additionally, the degree of injury is influenced by the forces generated upon impact.

Acetabular fractures are uncommon in the clinical setting [10–12]. Laird and Keating determined the overall incidence of such fractures as three fractures in 100,000 trauma patients per year [12]. They included all trauma admissions to the emergency department. Acetabular fractures mainly occur after high-energy trauma, e.g. motor vehicle accidents and falls from height [10–14]. Such incidents are known to cause poly-trauma and therefore have a high mortality rate. Fracture patterns could be indicative for trauma mechanism(s) [3]. In fatal accidents, this can have forensic importance, since information obtained from the postmortem (radiological) investigation of the body can be used for the detection and documentation of forensic evidence. Additionally, this information can be useful in the reconstruction of the incident.

**Fig. 1:**
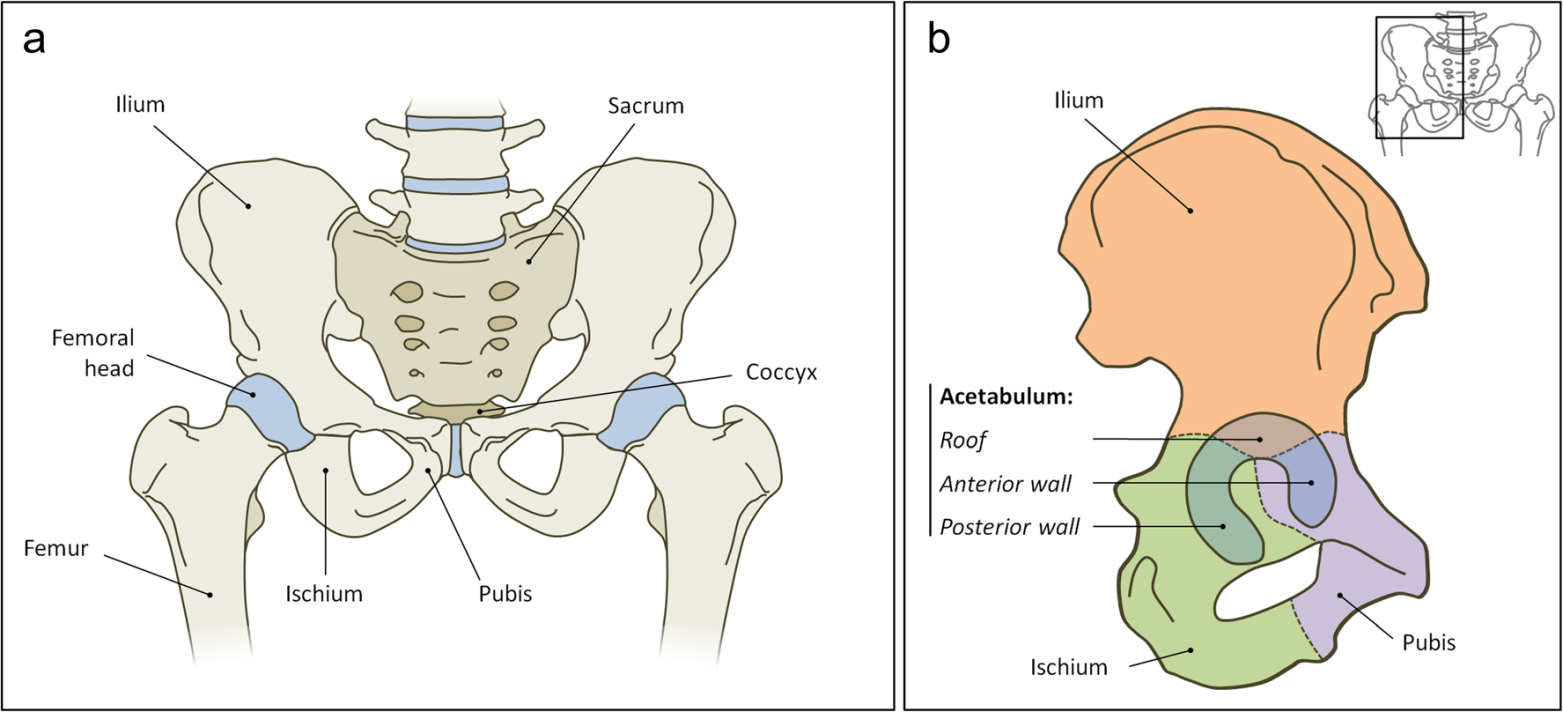
The anatomy of the pelvis **a** Ventral view of the pelvic region. Cartilaginous parts in blue and bony parts in grey. **b** Lateral view of the right hemi pelvis. The ilium (orange), ischium (green) and pubis (purple) all contribute to the acetabulum (blue). Schematic drawings by authors

Pelvic fractures or injuries in a forensic setting have been reported previously, e.g. after falls from height or civil aviation accidents [3–6]. The acetabulum is a complex three dimensional (3D) structure, which is a component of the pelvis (Fig. 1). It is anatomically important because the acetabulum connects the axial skeleton with the lower appendicular skeleton; the femoral head articulates with the cup-shaped acetabulum of the pelvis [10]. An acetabular fracture may occur when the femoral head transmits a force towards the acetabulum that was applied on the greater trochanter, knee or foot [13].

The purpose of this study was to describe acetabular fracture patterns after fatal small aircraft accidents and to investigate the relationship between the presence of fractures of the spine and/or lower extremities and an absence of acetabular fractures. We hypothesized that if there was extensive fracture of the lower extremities or the spinal column, the acetabulum would remain intact. Conventional radiographs and PMCT of the subjects in this retrospective study were obtained from a forensic radiological database [15].

## Materials and methods

### Study population

All cases from the Dutch forensic radiological database relating to fatal aviation accidents, that occurred between 2000 and 2016, were selected [15]. Starting in 2000, per a request of the pathologist in the Netherlands Forensic Institute (NFI), bodies (in body bags) were imaged at the Groene Hart Hospital (GHH) in Gouda, The Netherlands prior to autopsy. These images were subsequently analyzed by a forensic radiologist. The images, autopsy report and radiology report were later used to create the SPSS database (Armonk, NY, USA) [15]. For this retrospective study, all aviation accident cases were retrieved from the SPSS database and re- analyzed. General case information was obtained from the autopsy reports at the NFI and/or from the files of the Dutch Safety Board (Onderzoeksraad voor Veiligheid). Cases concerning commercial (*n* = 5) or military aviation accidents (*n* = 1) were excluded from the study.

A total of 35 fatalities in 23 aviation accidents were retrieved from the forensic radiological database [15]. After applying the above mentioned exclusion criteria, 29 victims of 20 small aircraft aviation accidents were included (Table 1). Among these victims, there were 27 males (93%) and two females (7%). The median age among the victims was 55 years, in a range from 21 to 76 years. We were unable to distinguish pilots from passengers, as many passengers were licensed to fly and therefore can be considered co-pilots who were actively involved in controlling the airplane.

**Table 1:** Overview of the victims in small aircraft accidents

Victim	Sex (age)	Fx	Crash site	Plane type	Info crash	Imaging
A1	M (26)	No	Water	New Piper PA-44-180	Graveyard spin, engine failure	X ray
A1.1	M (21)	No				
A1.2	M (21)	No				
A2	M (26)	No	Land	Aviat A-1 Husky	Steep nose down	X ray
A3	M (45)	No	Land	KP- 2UR Kappa Sova	Engine failure	X ray
A3.1	M (48)	Yes				
A4	M (39)	Yes	Land	Comco Ikarus C42B	Crash into house	X ray
A4.1	F (48)	Yes				
A5	M (72)	Yes	Land	Cessna 152	Bad weather	X ray
A5.1	M (60)	Yes				
A6	M (73)	Yes	Land	Air Creation Mild GT 582 ES	Crash into roof hangar	X ray
A7	M (64)	No	Land	Comco Ikarus C42B	Inflight collision banner	CT
A8	M (76)	Yes	Land	Scheibe SF25 C Falke	Uncontrolled movements	X ray
A9	M (40)	Yes	Land	Cessna 172R	Collision with other plane	X ray
A9.1	M (46)	Yes				
A10	M (56)	Yes	Water	Zenair CH601XL Zodiac	Break up wing in flight	X ray
A10.1	M (57)	Yes				
A11	M (61)	Yes	Land	Schleicher ASK-13	Problems during landing	CT
A12	M (54)	No	Land	Eurocopter EC 130 B4	Graveyard spin	CT
A13	M (28)	No	Land	Cessna 172	Problems banner pick- up	CT
A14	M (45)	No	Land	Yakolev Yak- 52	Steep nose down	CT
A15	M (54)	No	Land	Comco Ikarus C42	Steep nose down	CT
A16	M (50)	Yes	Land	General Avia F.22B	Steep nose down	CT
A17	M (36)	Yes	Land	Diamond DA-40D TDI	Collision other plane	CT
A17.1	F (23)	Yes				
A18	M (71)	No	Water	Cessna 172P	Touched water surface in descent	CT
A19	M (24)	Yes	Land	Extra EA- 300 L	Aerobatics gone wrong	CT
A19.1	M (26)	Yes				
A20	M (76)	Yes	Water	Cirrus SR20	Bad weather	CT

*M* male, *F* female, *Fx* fracture of clinical acetabulum, *CT* CT scans, *X ray* conventional radiographs

### Imaging

Conventional radiographs were captured with two Carestream X-ray systems: recent cases with Carestream Evolution and older cases from before 2009 with Carestream 7500 (Carestream Health Netherlands B.V., Eemnes, The Netherlands). Total body PMCT scans were obtained through scanning with three different scanners: Toshiba Aquilon 64 slice for cases since 2009 (Toshiba Medical Systems Europe B.V., Zoetermeer, The Netherlands) and the Toshiba Aquilon 32 slice and Siemens Somatom 4 (Siemens Harmony, Siemens, Erlangen, Germany) for older cases. The imaging protocol for the pelvis (and lower extremities) was 120 kV/363 mA/1 s rotation/0.5 mm slice thickness /reconstruction 0.5 mm.

Radiographs and CT scans were re-analyzed in Carestream picture archiving and communication system (PACS) (Carestream Health Netherlands B.V., Eemnes, The Netherlands). Three dimensional reconstructed images were available in PACS for all PMCT cases. Fractures of the acetabulum and adjacent structures (i.e. pelvis, lower extremities and spinal column) were scored using an in-house developed case report form. All images were scored by one forensic radiologist, with 17 years of experience in forensic radiology (HdB), blinded to the autopsy reports, and an independent research assistant (MT).

### Anatomical definitions

Judet and Letournel set up the first acetabular fracture classification system in 1964 [13, 14]. Additionally, they explained the mechanics of acetabular fractures to better understand how such injuries could be caused and treated [16]. In the decades that followed other clinicians and researchers like Harris et al. assessed acetabular fractures [10], with an aim to simplify their classification. However, the Letournel classification system is clinically most often used for its application in conventional radiographs as well as in CT scans [10].

**Fig. 2:**
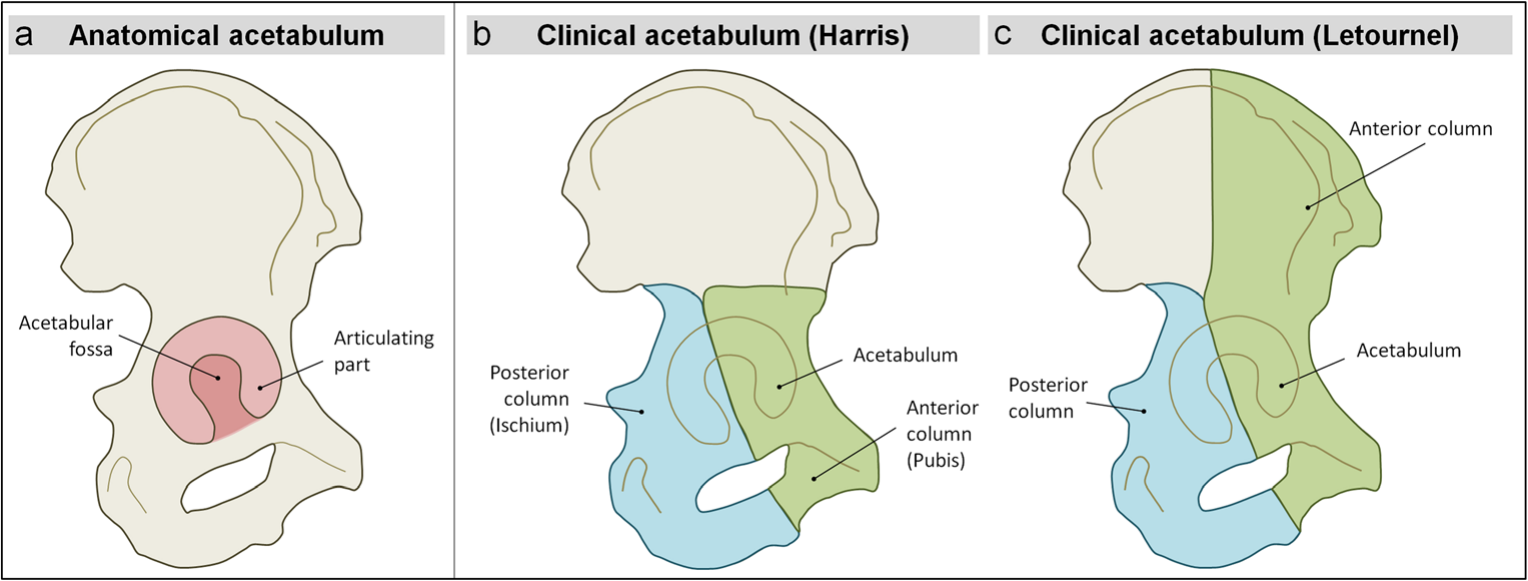
Anatomical definitions depicted on a right hemi pelvis, lateral view **a** Anatomy of the anatomical acetabulum (light pink) and its acetabular fossa (darker pink). **b** Anatomy of the clinical acetabulum as described by Harris including anterior and posterior column (green and blue respectively). **c** Anatomy of the clinical acetabulum according to the Letournel classification system. Note that the anterior column includes the medial half of the ilium bone for clinical implications. Schematic drawings by authors

For this retrospective study, in which we strived to describe acetabular fracture patterns in victims of aviation accidents, a distinction has been made between the anatomical acetabulum and the clinical acetabulum. The anatomical acetabulum was considered to be composed of the anterior wall, posterior wall, and roof, i.e. the articulating part of the acetabulum with the femoral head [10]. The central region where the femoral head did not articulate with the acetabulum was defined as the acetabular fossa. This part of the anatomical acetabulum is the location for ligament attachment (Fig. 2) [10].

The clinical acetabulum is composed of the anatomical acetabulum, an anterior column (pubis), and a posterior column (ischium) [17, 18]. In this case series, we distinguished between fractures in the anatomical acetabulum and fractures in the clinical acetabulum. As there were no clinical implications in this study, the classification of fractures was kept simple, following the Harris classification instead of using the thorough, though complicated, Judet and Letournel classification system [17, 18], because the Judet and Letournel classification system was created to increase the prognosis of healing of acetabular fractures, based on different surgical approaches [13, 14]. This case series was solely concerned with postmortem images and hence treatment of the fractures was out of scope.

### Statistical analyses

Case report forms were used to score and collect the data; Microsoft Excel 2010 (Microsoft Corporation, Redmond, WA, USA) was used for the processing of the data. Statistical analyses were performed with IBM SPSS Statistics 24 and Microsoft Excel 2010. To test independence between fracture location and other variables, the Chi- square test was used; *p* < 0.05 was considered to be statistically significant.

## Results

The pelvis was intact in eight victims; 21 of the 29 victims showed pelvic injuries. Twelve of 29 victims had a diastasis of the pubic symphysis or the sacroiliac joint, in combination with a fracture of the pelvis as can be expected by interruption of the pelvic ring. This case series showed 19 out of 29 victims (66%) had fractures of the clinical acetabulum, including the anterior column (pubis) and posterior column (ischium), as presented in Table 2. Eleven victims had bilateral acetabular fractures, whereas eight victims exhibited only unilateral fractures. Left-sided fractures (16/29) were more prevalent than right-sided fractures (14/29). When considering the left and right acetabulum as two separate entities, 40 out of 58 studied clinical acetabulums and 12 out of 58 anatomical acetabulums were fractured. The anatomical acetabulum, thus the walls, roof and acetabular fossa, was fractured in 8 out of 29 victims (28%). Unilateral fractures of the anatomical acetabulum (*n* = 6) were more prevalent than bilateral fractures (*n* = 2).

**Table 2:** The number of victims with fractures per location, including total percentages

	Total (*n* = 29)	Percentages of 29 (%)
Clinical acetabulum		
Fractures	19	66
Bilateral	11	38
Left	5	17
Right	3	10
None	10	34
Anterior column		
Bilateral	10	35
Left	4	14
Right	4	14
Posterior column		
Bilateral	10	35
Left	5	17
Right	3	10
Anatomical acetabulum		
Fractures	8	28
Bilateral	2	7
Left	2	7
Right	4	14
None	21	72
Anterior wall		
Bilateral	1	3
Left	0	0
Right	5	17
Posterior wall		
Bilateral	0	0
Left	3	10
Right	2	7
Roof		
Bilateral	0	0
Left	0	0
Right	4	14
Acetabular fossa		
Bilateral	1	3
Left	2	7
Right	2	7

### Acetabular column fractures

**Fig. 3:**
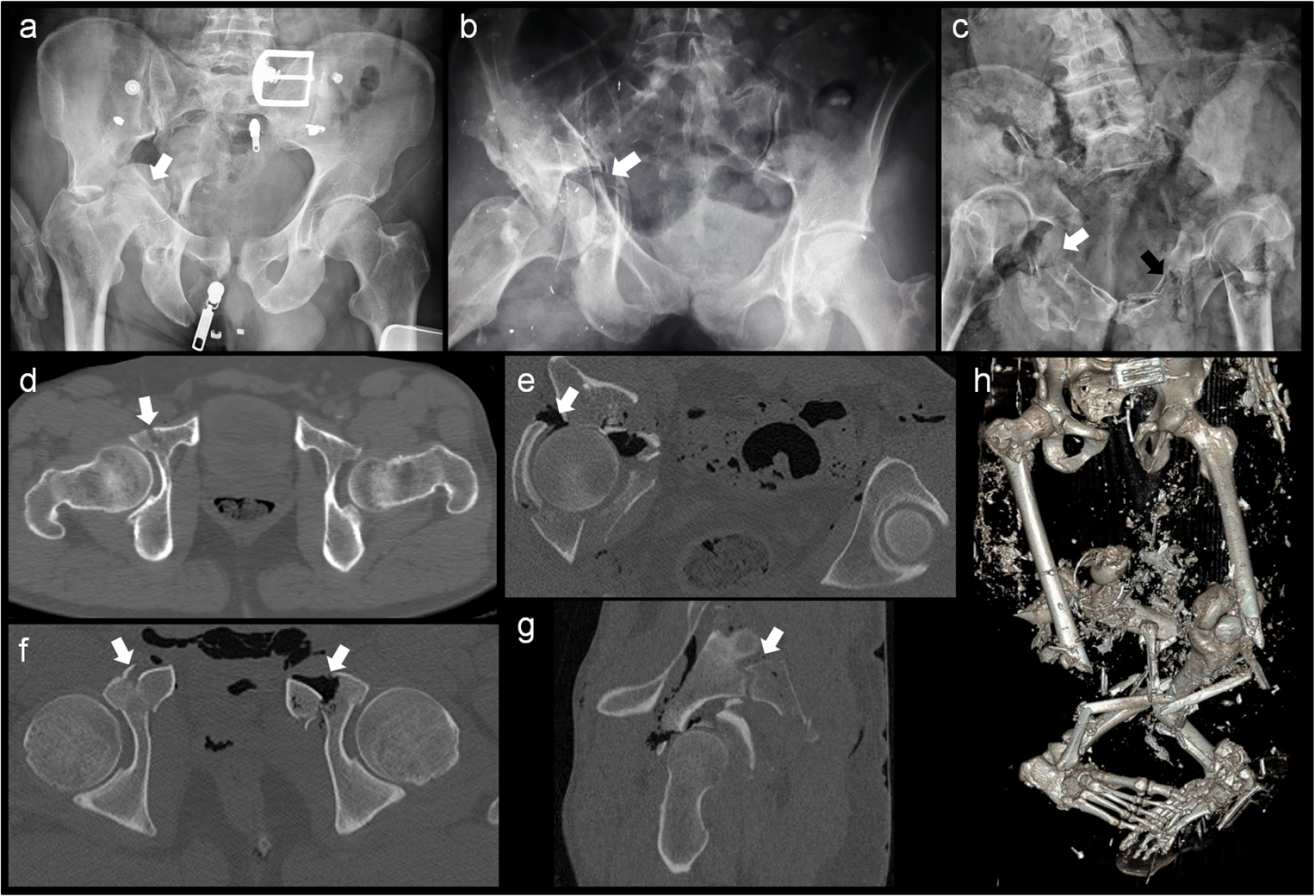
Examples of radiological images of acetabular fractures **a** Conventional radiographic anterior-posterior (AP) view of the pelvis. Right-sided acetabular fracture with medial directed penetration of the femoral head through the acetabulum (white arrow). **b** Conventional radiographic AP view of the pelvis. Both anterior and posterior column were fractured on the right side (white arrow). **c** Conventional radiographic AP view of the pelvis. Bilateral extensive fracture of both anterior and posterior columns (white and black arrows). **d** Transversal CT view of a subtle right-sided anterior column fracture (white arrow). **e** Transversal CT view of the pelvis. Right-sided comminuted anatomic acetabular fracture with penetration of the femoral head through the acetabulum (white arrow). **f** CT transversal view of a bilateral anterior column fracture (white arrows). **g** The same case as in f. Additional CT lateral view of the right hemi pelvis with comminution of the acetabulum. Note the acetabular roof fracture (white arrow). **h** 3D–CT reconstruction of the pelvis and lower extremities. The telescopic fracture pattern of the lower extremities is typical for aviation accidents [15]. Note also multiple pelvic fractures and diastasis of the symphysis in contrast to the seemingly intact pelvic region of the body (Fig. 4e)

Anterior acetabular column fractures occurred in 18 out of 29 victims; 14 sustained a left-sided anterior column fracture, and 14sustained a right-sided fracture. An example of a right-sided anterior column fracture can be seen in Fig. 3b and d. Ten of the 18 victims with anterior column fractures exhibited bilateral anterior column fractures (Fig. 3c and f).

Eighteen of the 29 victims also showed posterior column fractures, however these 18 victims were not the same 18 victims with anterior column fractures. A total of 15 victims portrayed left-sided posterior column fractures, whereas 13 portrayed right-sided fractures. Of these 18 victims with posterior column fractures, there were 10 victims with bilateral posterior column fractures.

### Acetabular wall fractures

The overall frequency of anterior wall fractures in the study population was 6 out of 29 (Table 2). No isolated left-sided anterior wall fractures occurred, there was one victim with a bilateral anterior wall fracture and five victims who showed a right-sided anterior wall fracture. A right-sided posterior wall fracture occurred twice, whereas left-sided posterior wall fractures three times. None of the victims exhibited a bilateral posterior wall fracture.

### Acetabular roof and acetabular fossa fractures

The acetabular roof was fractured on the right side in 4 out of 29 victims, with penetration of the femoral head in two victims (Fig. 3a, e and g). The right-sided acetabular fossa was fractured in two victims. Isolated left-sided fractures of the acetabular fossa occurred in two victims. One out of 29 victims had a bilateral acetabular fossa fracture.

### Fractures in the lower extremities and spinal column

Fractures of the lower extremities occurred in 25 out of 29 victims (Fig. 3h). The number of victims who portrayed one or more fractured extremities in combination with an acetabular fracture was 16 out of 29. There were nine victims with fractures in the lower extremities, without a fracture in the acetabulum. Statistical analysis did not reveal a significant dependence of the prevalence of lower extremity fractures on the absence of acetabular fractures (*p* = 0.667).

Regarding the spinal column, fractures were encountered in 21 of the 29 victims. Seven victims had one or more fractures in the spinal column without the presence of an acetabular fracture. In 14 victims, a fracture of both the spinal column and acetabulum occurred. There was no significant dependence of the prevalence of fractures in the spinal column on the absence of acetabular fractures (*p* = 0.833).

## Discussion

We found that two thirds of the victims of fatal aviation accidents in small aircrafts in our case series had uni- or bilateral acetabular fractures. The prevalence of acetabular fractures is still 28% when solely considering the anatomical acetabulum. The high number of acetabular fractures in this population is remarkable. However, due to the lack of publications describing these complex and high energetic trauma mechanisms, comparison with other studies is not possible.

It would be interesting to see if the 34% of victims who did not have any fractures of the acetabulum sustained lesions outside the acetabular region, hence the assessment of fractures in the lower extremities (femur, tibia and fibula) and spinal column. Depending on the impact force and direction of the force transmitting through the bones, some body parts might be spared while different body parts suffered extensive (skeletal) injuries. There was no significant dependence between fractures of the lower extremities and fractures of the acetabulum. The radiological images did reveal many telescoping fractures of the lower extremities, meaning that the long bones of the lower extremities are comminuted and shortened. Additionally, fractures of the spinal column in relation to acetabular fractures also did not reveal a significant dependence. Therefore, the direction of the impact force was substantial enough to cause fractures in multiple skeletal regions.

Judet and Letournel discussed that fractures of the anterior column of the acetabulum can occur when the greater trochanter of the femur experiences a force [16]. This theoretical explanation means that victims with anterior column fractures experienced a lateral force to the greater trochanter upon impact. A fracture of the anterior column could therefore happen when the aircraft crashes on one of its sides. As for posterior column fractures, fracturing can occur when the bended knee experiences a ventral force and transmits this through the femur into the pelvis [16], which could suggest that the aircraft crashed on its nose. However, ranges of flexion or extension and abduction or adduction can create different fracture patterns. Any attempt to reconstruct the mechanism of a complex accident like an aircraft accident requires more than the analysis of the fracture pattern of a single fracture and no conclusions should be drawn regarding accident reconstruction based on the pattern of acetabular fractures alone. Also important to note is that the biomechanical properties of bone fracture are complicated and depend on many different factors, such as age and gender of the victim. Due to the retrospective nature of the current study, it was not possible to assess the bone properties of each of the victims.

**Fig. 4:**
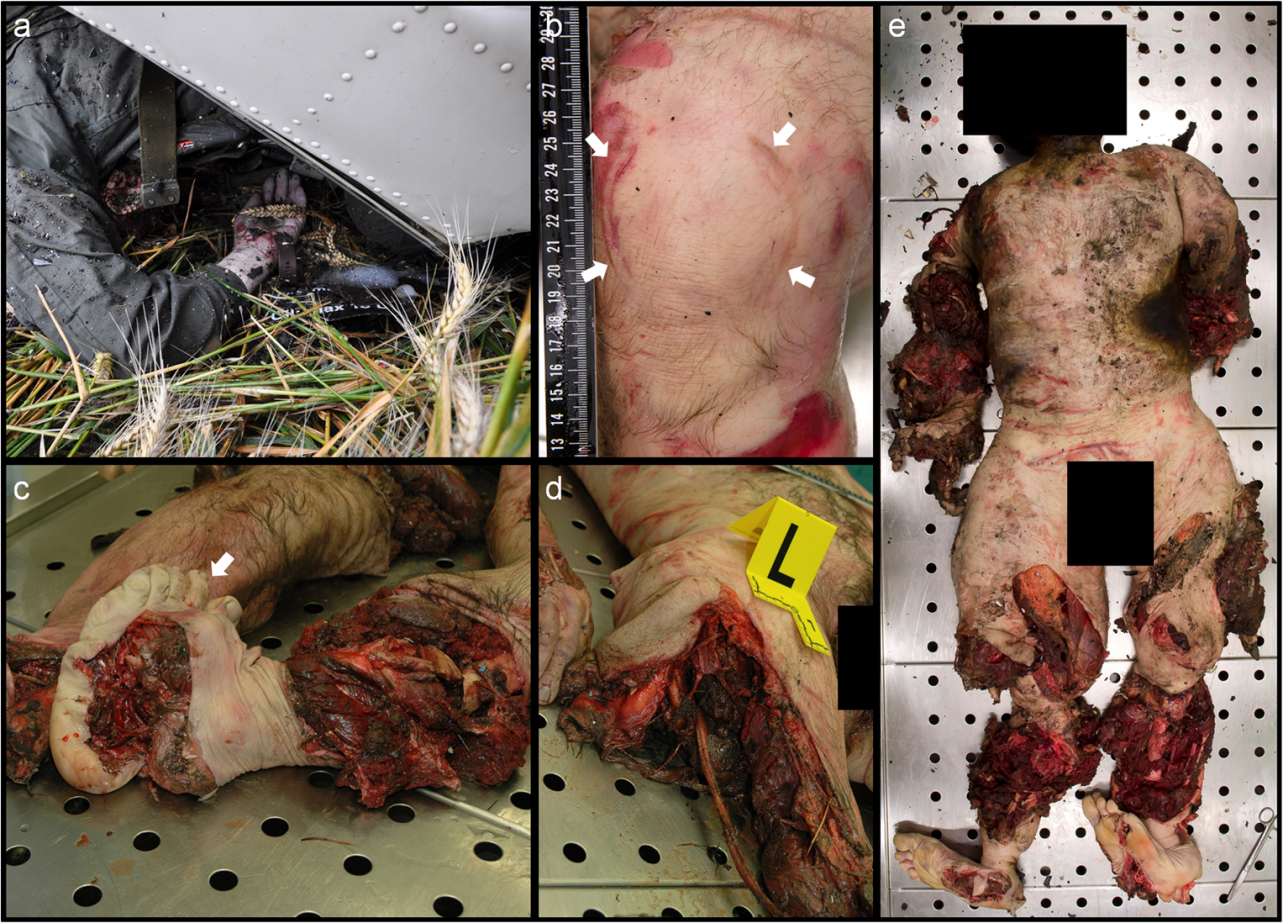
Images of various victims from small aircraft crashes **a** Picture of a detail of the victim, taken at the scene of the crash. The hand and part of the aircraft are recognizable. **b** Ventral view of a bended knee. Note the round traumatic impression (arrows), most likely caused by hitting the dashboard. **c** Extensive destruction of the left lower leg and right upper extremity and both feet. The toes of the left foot are recognizable (arrow). **d** A large laceration of the right upper extremity. **e** Ventral total body view. Note the destruction of all four extremities in contrast to the seemingly intact pelvic region. Typical telescopic shortening of the legs [15]. A 3D–CT scan of this victim is shown in Fig. 3h

In fractures with a posterior wall component, the most common fracture mechanism is trauma to the distal femur or knee (Fig. 4b) while the hip is abducted, e.g. a knee hitting the dashboard in a car upon impact [10, 13, 16]. In the clinic, fractures with posterior wall component occur most often [14]. Interestingly, only five victims sustained a posterior wall fracture. Small aircrafts also have dashboards and therefore the mechanism of fracture was expected to be similar to those in cars. The low number of posterior wall fractures suggests that impact mechanisms in an aviation accident differ from those in a motor vehicle accident [10, 11, 16]. Additionally, despite the high impact forces in aviation accidents, we observed a low number (*n* = 2) of femoral head penetrations. As already mentioned above, the direction of forces seems to be more diverse (and complicated) in aviation accidents compared to motor vehicle accidents. This idea is also supported by our high number of bilateral acetabular fractures. Eleven of the 19 victims with acetabular fractures exhibited bilateral fractures. This observation could indicate that the impact force and direction was sufficiently extensive to fracture the acetabulum in both hemi pelvises.

Since Judet and Letournel set up their classification system in 1964, radiographic technology has improved. The use of CT scans and their 3D reconstruction possibilities has resulted in improved interpretation of the anatomical complexity of the pelvis, which was also pointed out by Geijer and El- Khoury [11], and therefore more accurate identification of traumatic injuries in this region is possible. In our study, the assessment of acetabular fractures in 16 victims imaged before 2008 was solely based on conventional radiographs taken in the anteroposterior direction. Judet and Letournel suggested the use of two additional oblique views of the affected hemi pelvis [13, 14]. But assessment of those conventional radiographs remained limited compared to the CT scans performed nowadays. Therefore, assessment should be done solely based on CT scans to ensure the highest accuracy in clinical or post-mortem settings [15]. The strength of the whole body PMCT is that it allows for the analysis of whole body fracture patterns. Following this, a multi-slice PMCT scan has to be performed prior to autopsy to accurately access all body fractures and in particular pelvic fractures, since the pelvic region often appears relatively intact during autopsy, when compared to other body regions (Fig. 4).

In summary, this is the first detailed assessment of acetabular fractures in victims of fatal aviation accidents. The results of this case series show the high prevalence of fractures in different acetabular locations, with anterior and posterior column fractures exhibited most often. Fracture of the acetabulum did not show significant dependence on fracture of the lower extremities or the spinal column. The use of PMCT scans for the assessment of skeletal injuries after aviation accidents is recommended in addition to autopsy, as autopsy does not give complete insight in skeletal lesions.

## Key points

1. In victims of small aircraft aviation accidents acetabular fractures were frequently encountered.
2. Bilateral fractures happened often; 11 of the 19 victims exhibited bilateral acetabular fractures.
3. The absence of acetabular fractures is statistically independent from fractures of the leg or spinal column.
4. A postmortem CT scan should be performed prior to autopsy to accurately access pelvic fractures.
5. Postmortem CT scans are preferred over conventional radiographs in the assessment of pelvic injuries after aviation trauma.
